# Patterns of Ambulatory Medical Care Utilization and Rheumatologist Consultation Predating the Diagnosis of Systemic Lupus Erythematosus: A National Population-Based Study

**DOI:** 10.1371/journal.pone.0101485

**Published:** 2014-07-07

**Authors:** Ning-Sheng Lai, Tzung-Yi Tsai, Malcolm Koo, Kuang-Yung Huang, Chien-Hsueh Tung, Ming-Chi Lu

**Affiliations:** 1 Division of Allergy, Immunology and Rheumatology, Dalin Tzu Chi Hospital, Buddhist Tzu Chi Medical Foundation, Chiayi, Taiwan; 2 School of Medicine, Tzu Chi University, Hualien, Taiwan; 3 Department of Medical Research, Dalin Tzu Chi Hospital, Buddhist Tzu Chi Medical Foundation, Chiayi, Taiwan; 4 Department of Nursing, Tzu Chi College of Technology, Hualien, Taiwan; 5 Department of Environmental and Occupational Health, College of Medicine, National Cheng Kung University, Tainan, Taiwan; 6 Dalla Lana School of Public Health, University of Toronto, Toronto, Ontario, Canada; Center for Rheumatic Diseases, India

## Abstract

**Objective:**

To investigate the records of ambulatory medical care from patients predating the diagnosis of systemic lupus erythematosus (SLE) using nationwide, population-based claims data.

**Methods:**

The frequencies and costs of ambulatory medical care utilization in 337 newly-diagnosed SLE cases between 2004 and 2010, identified from Taiwan's National Health Insurance Research Database, were compared with 1,348 controls who were frequency matched for sex, age, and the catastrophic illness certificate application year of the cases.

**Results:**

Patients with SLE had a median frequency of ambulatory medical care utilization compared with controls one year prior to the index date (22 vs. 2, *P*<0.001). The differences were significant throughout all eight annual periods. Similarly, the inflation-adjusted costs of ambulatory medical care utilization in patients with SLE increased annually over the study period, from a median of US$18 eight years prior to the index date to US$680 one year prior to the index date. Diseases of the respiratory system (International Classification of Diseases, Ninth Revision, Clinical Modification [ICD-9-CM] codes 460–519), digestive system (ICD-9-CM codes 520–579), musculoskeletal system and connective tissue (ICD-9-CM codes 710–739, excluding 710.0), and skin and subcutaneous tissue (ICD-9-CM codes 680–709) were the top four common causes of visits in the 0.5 to 2 year period preceding the index date and percentages of SLE patients suffered from these disorders increased progressively over the study period. Only 56.4% of the patients with SLE had consulted a rheumatologist and most of the serology tests were done within one year predating the index date.

**Conclusions:**

Increased frequencies and costs of ambulatory care utilization among Taiwanese patients with SLE occurred several years predating their definitive SLE diagnosis. When multisystemic disorders are presented in young female patients, the possibility of SLE should be considered and screened with tools such as the antinuclear antibody test.

## Introduction

Systemic lupus erythematosus (SLE) is a complex systemic autoimmune disease with a wide range of clinical features that involve almost every organ systems in the body. The peak age of onset is around late teens and early 40 s with a women-to-men ratio of 9∶1. In addition, people of African or Asian descent are at particular risk for developing SLE and exhibit more severe clinical manifestations compared with those of European descent [1]. Genetic, epigenetic, environmental, hormonal, and immunoregulatory factors all contribute to the development of SLE [2].

SLE is notoriously challenging to diagnose because of its heterogeneous clinical presentation and the lack of good biomarkers [3]. Although SLE is characterized by the presence of several autoantibodies [4], [5], there exists a long preclinical phase from the development of autoimmunity to its clinical onset [6]. The gradual sequential development of autoantibodies takes many years preceding the clinical onset of SLE [6]–[9]. Moreover, clinical criteria of SLE, including arthritis and serosititis, skin, hematological system and renal disorder, are also noted several years predating the diagnosis of SLE [4], [7], [10]. In addition, the severity of the SLE clinical manifestations during the pre-SLE period can fluctuate and many laboratory tests and imaging studies are often needed before a definitive diagnosis of SLE can be made. For these reasons, general practitioners may not suspect the early phase of SLE in their patients and delay a referral to a rheumatologist. Therefore, it is critical to assess the preclinical phase of SLE. However, due to the low incidence of SLE and its highly variable initial manifestations, it is highly inefficient and difficult to assess the preclinical phase of SLE using a cohort study design. Although a case control design is more efficient and feasible to conduct, the results are highly susceptible to recall bias in patients with SLE. Hence, we used an alternative approach based on the claim data of ambulatory medical care to investigate the general health conditions among patients predating the diagnosis of SLE.

Our previous study showed that the frequencies and costs of ambulatory visits in patients gradually increased as the time approaching the diagnosis of rheumatoid arthritis [11]. Yet, to our knowledge, no studies have reported the clinical disorders in a systematic manner among patients with SLE predating the diagnosis of SLE using population-based data. Therefore, the aim of this study was to investigate the pattern of ambulatory medical care utilization over an eight-year period in patients predating the diagnosis of SLE using a nationwide, population-based claim database.

## Patients and Methods

### Study design

This was a retrospective comparison study using nationwide, population-based cohort data.

### Data sources

This study is based on the Longitudinal Health Insurance Database 2000 (LHID2000) of the Taiwan's National Health Insurance Research Database (NHIRD), provided by the Taiwan National Health Research Institute. LHID2000 contains comprehensive utilization and enrollment information from 1996 to 2010 for a randomly selected sample of one million beneficiaries registered in 2000, representing approximately 5% of Taiwan's population. The NHIRD consists of de-identified secondary data of inpatient and ambulatory medical care services of all enrollees of the National Health Insurance (NHI) scheme. The NHI is a single-payer compulsory health insurance program instituted in 1995 and covers over 99% of the Taiwan's population [12].

Under the NHI scheme, insured individuals afflicted with any of the 30 categories of catastrophic illness, specified by the Bureau of NHI, can apply for catastrophic illness certificates. If approved, these patients are exempted from co-payments when seeking medical care related to their catastrophic illness. The issuance of certificates is validated through careful review of medical records, laboratory studies, and imaging studies by at least two specialists.

The study protocol was reviewed and approved by the institutional review board of the Dalin Tzu Chi Hospital, Buddhist Tzu Chi Medical Foundation (No. B10104020). Since the NHIRD files contain only de-identified secondary data, the need for informed consent from individual subjects was waived.

### Study subjects

Cases of SLE were identified from the catastrophic illness patients file of the NHIRD with corresponding International Classification of Diseases, Ninth Revision, Clinical Modification (ICD-9-CM) code 710.0. Review of application of catastrophic illness certificates by the Bureau of NHI is based on the 1997 American College of Rheumatology (ACR) revised criteria for the classification of SLE [13]. We excluded patients under the age of 18 years and those with a catastrophic illness certificates issued for autoimmune diseases other than SLE. The date of application of catastrophic illness certificates in approved patients was considered as the index date in the analysis. For each SLE case, four controls were randomly selected from the LHID2000 with frequency-matching for 10-year age interval, sex, and the year of the index date (index year). The index date for each of the control was assigned by using the date of a randomly selected ambulatory visit for a given index year. A total of 337 SLE patients and 1,348 controls were included in the data analysis.

### Study variables

In this study, the costs of ambulatory medical care including physician consultation fees (Western medicine, dentistry, and traditional Chinese medicine), visits to emergency departments, medical procedures, pharmacy expenditure, laboratory diagnosis tests, imaging examination, and rehabilitation therapy, were charged according to the payment schedule of the NHI. All cost figures were converted to US dollars based on annual exchange rates and were further adjusted to 2010 dollars using Taiwan's consumer price index to take into the account of inflation.

The study subjects were retrospectively traced back up to eight years preceding the index date when examining the frequencies and costs of ambulatory medical care utilization. The frequencies and costs of the ambulatory medical care utilization on the index date itself were excluded from the analysis. The main causes of the ambulatory medical care utilization were defined by the use of principal diagnosis of the ICD-9-CM coding system [14]. In addition, we obtained frequencies of hospitalization of the study subjects from the LHID2000 with discharged day prior to the index date. Information on whether medical tests including antinuclear antibodies (ANA) via indirect immunoflorescence assay, complete blood count (CBC), alanine aminotransferase (ALT), creatinine (Cre), and chest X-ray had been performed on the study subjects were also obtained from the LHID2000. The geographic location in which the subjects resided were categorized into north, central, south, and east regions. The Deyo adaptation of the Charlson Comorbidity Index (CCI) [15] was used to quantify the subject's comorbidity patterns based on their medical records six months prior to the beginning of the eight-year study period.

### Statistical analysis

Data were expressed as mean and standard deviation (SD) or median with interquartile range, as appropriate. Chi-square test and Wilcoxon rank-sum test were used to compare the characteristics between patients with SLE and controls. The frequencies and costs of ambulatory medical care utilization between the two groups were compared using Wilcoxon rank-sum test.

Trends in the frequencies of ambulatory medical care utilization across the eight annual periods were evaluated using generalized estimating equations (GEE) with Poisson log-linear link function. GEE was used to take into the account the statistical dependency of multiple observations for each subject over time. Incident rate ratios were estimated with GEE using the frequency at eight year preceding the index date as the reference category. Trends in the costs of ambulatory medical care utilization were evaluated using GEE with log link function with gamma distribution. Cost ratios were estimated using the cost data at eight year preceding the index date as the reference category. Analyses of frequencies and costs of medical care utilization using GEE were adjusted for age, sex, and CCI. All statistical analyses were conducted using SAS statistical software, version 9.2 for Windows (SAS Institute Inc., Cary, USA). A *P*<0.05 was considered statistically significant.

## Results

### Demographic data of patients with SLE and controls

Since age and sex were frequency matched between cases and controls, no significant differences were observed in these two variables between the cases and controls. In addition, no significant differences were observed in geographical regions and CCI between cases and controls (Table 1).

**Table 1:** Characteristics of the patients with systemic lupus erythematosus and controls.

		Group, mean ±SD or number (%)		*P*
		SLE	Controls	
		n = 337	n = 1,348	
**Age at enrollment (years)**		37.4±18.2	37.4±18.1	0.997
**Sex (% female)**		293 (86.9)	1159 (86.0)	0.646
**Geographical region**				0.123
	North	152 (45.1)	413 (50.4)	
	Central	88 (26.1)	207 (25.3)	
	South	90 (26.7)	175 (21.4)	
	East	7 (2.1)	24 (2.9)	
**Charlson Comorbidity Index**		0.09±0.39	0.05±0.38	0.947

SD, standard deviation; SLE, systemic lupus erythematosus.

### Frequency and cost of ambulatory medical visits

We found that SLE patients, prior to diagnosis of SLE, used ambulatory medical services more frequently compared with controls (Table 2). Even after tracing back as far as eight years before the index date, there was still a significant higher median frequency of ambulatory visits in patients with SLE compared with controls (1 vs. 0, *P*<0.001). The differences in median frequency of ambulatory visits gradually increased as the period approached the index date. The differences in the median frequency of ambulatory visits between the patient with SLE and controls became largest (22 vs. 2, *P*<0.001) one year prior to the index date.

**Table 2:** Frequencies and costs of ambulatory medical care utilization in patients with systemic lupus erythematosus and controls over an eight-year period preceding the index date.

Years preceding index date	Annual frequencies of ambulatory medical care utilization		*P*	Annual costs of ambulatory medical care utilization		*P*
	Patients with SLE	Controls		Patients with SLE	Controls	
	n = 337	n = 1,348		n = 337	n = 1,348	
8	8.31±13.23	4.04±9.63	<0.001	232±697	98±454	<0.001
	(1, 0–13)	(0, 0–3)		(18, 0–251)	(0, 0–47)	
7	9.74±12.80	4.95±10.76	<0.001	289±1450	107±288	<0.001
	(5, 0–15)	(0, 0–6)		(82, 0–293)	(0, 0–94)	
6	12.15±13.64	6.05±11.79	<0.001	398±1488	135±407	<0.001
	(8, 1–18)	(0, 0–9)		(142, 13–407)	(0, 0–145)	
5	14.41±13.18	7.30±12.74	<0.001	520±2000	186±848	<0.001
	(11, 5–20)	(1, 0–10)		(218, 73–453)	(12, 0–204)	
4	16.03±13.61	7.86±12.45	<0.001	632±2404	208±903	<0.001
	(13, 6–23)	(2, 0–12)		(243, 93–542)	(16, 0–225)	
3	16.81±13.27	8.10±12.83	<0.001	750±2420	216±893	<0.001
	(14, 6–23)	(2, 0–12)		(265, 110–632)	(20, 0–220)	
2	17.47±15.18	7.84±12.72	<0.001	760±2460	218±949	<0.001
	(13, 6–25)	(1, 0–12)		(291, 109–751)	(27, 0–230)	
1	25.23±15.24	8.07±13.05	<0.001	1134±2503	216±897	<0.001
	(22, 15–32)	(2, 0–12)		(680, 419–1186)	(27, 0–230)	

Values are mean ± standard deviation and (median, 25^th^–75^th^ percentile).

Costs were converted to US dollars based on annual exchange rates and adjusted to 2010 figures based on Taiwan's consumer price index.

SLE, systemic lupus erythematosus.

Similarly, the inflation-adjusted costs of ambulatory health care utilization were significantly higher in patients with SLE compared with controls predating index date. Compared with the controls, the median cost of annual ambulatory visits was significantly higher in patients with SLE (US$18 vs. US$0, *P*<0.001) eight years prior to the index date. The differences between the two groups gradually increased as the time approaching the diagnosis of SLE and became largest in the year just before the index date (US$680 vs. US$27, *P*<0.001) (Table 2).

We observed significant trends of higher frequencies of visits over the eight-year study period in both patients with SLE and controls. However, the changes over time were more prominent in patients with SLE compared with controls as shown by the larger magnitude of the slope (β = 0.10 vs. β = 0.04) (Table 3). Regarding the costs of ambulatory medical utilization, significant trends of higher costs were also observed in both patients with SLE and controls. The changes over time was greater in patient with SLE compared with controls (β = 0.20 vs. β = 0.06) (Table 4). Over the eight-year period, the annual costs of ambulatory care visits showed nearly a quadruple increase in patients with SLE but only elevated 49% in controls.

**Table 3:** Trends in annual frequencies of ambulatory medical care utilization over an eight-year period preceding the index date.

Years preceding index date	Patients with SLE				Controls			
	*β* IRR 95% CI *P*				*β* IRR 95% CI *P*			
8	Reference year				Reference year			
7	0.02	1.02	0.89–1.16	0.746	0.04	1.04	0.95–1.13	0.346
6	0.09	1.09	0.96–1.24	0.187	0.11	1.11	1.02–1.22	0.028
5	0.13	1.14	0.99–1.30	0.052	0.20	1.22	1.12–1.33	0.013
4	0.20	1.22	1.07–1.41	0.004	0.25	1.28	1.17–1.42	0.001
3	0.25	1.28	1.12–1.47	<0.001	0.26	1.29	1.18–1.42	<0.001
2	0.28	1.32	1.15–1.52	<0.001	0.27	1.31	1.19–1.44	<0.001
1	0.63	1.96	1.64–2.15	<0.001	0.29	1.34	1.22–1.48	<.001
	*β* = 0.10; trend test *P*<0.001				*β* = 0.04; trend test *P* = 0.001			

Estimates were adjusted for age, sex and Charlson Comorbidity Index using generalized estimating equation with a Poisson log-linear function.

SLE, systemic lupus erythematosus; IRR, incident rate ratio; 95% CI, 95% confidence interval.

**Table 4:** Trends in annual costs of ambulatory medical care utilization over an eight-year period preceding the index date.

Years preceding index date		Patients with SLE				Controls			
		*β* Cost ratio 95% CI *P*				*β* Cost ratio 95% CI *P*			
8		Reference year				Reference year			
7		0.20	1.21	0.86–1.77	0.286	0.05	1.00	0.93–1.19	0.940
6		0.44	1.55	1.09–2.26	0.014	0.08	1.08	0.90–1.29	0.415
5		0.59	1.81	1.10–3.01	0.013	0.26	1.29	1.06–1.58	0.009
4		0.79	2.21	1.43–3.39	<0.001	0.32	1.38	1.14–1.67	0.001
3		0.93	2.53	1.66–3.69	<0.001	0.37	1.44	1.20–1.74	<0.001
2		0.96	2.58	1.76–3.89	<0.001	0.39	1.49	1.22–1.84	<0.001
1		1.39	3.92	2.90–5.30	<0.001	0.40	1.49	1.23–1.80	<0.001
	*β* = 0.20; trend test *P*<0.001					*β* = 0.06; trend test *P* = 0.013			

Estimates were adjusted for age, sex and Charlson Comorbidity Index using generalized estimating equation with log link function and gamma distribution. Costs were converted to US dollars based on annual exchange rates and adjusted to 2010 figures based on Taiwan's consumer price index.

SLE, systemic lupus erythematosus; 95% CI, 95% confidence interval.

### Principal causes of ambulatory medical care utilization during the 0.5 to 2 years preceding the index date

To investigate the early and subtle clinical disorders before the apparently onset of SLE, we compared the principle causes of ambulatory medical visits in patients with SLE and controls in the 0.5 to two years period before the index date. We found that ambulatory visits were significantly higher in almost all categories of clinical disorders involving various organ systems in patients with SLE compared with controls. The top four common diseases categories leading to ambulatory medical visits were diseases of the respiratory system (ICD-9-CM codes 460–519), digestive system (ICD-9-CM codes 520–579), musculoskeletal system and connective tissue (ICD-9-CM codes 710–739, excluding 710.0), and skin and subcutaneous tissue (ICD-9-CM codes 680–709) (Table 5).

**Table 5:** Causes of ambulatory medical care utilization in patients with systemic lupus erythematosus and controls during the period 0.5 to 2 years preceding the index date.

Diagnosis (ICD-9-CM code)		Group, mean ±SD		*P*
		Patients with SLE	Controls	
		n = 337	n = 1,348	
**Diseases of the respiratory system (460–519)**		7.24±9.70	3.66±7.20	<0.001
	Acute respiratory infections (460–466)	5.78±7.06	3.09±6.35	<0.001
	Other diseases of the upper respiratory tract (470–478)	0.67±2.09	0.31±1.52	<0.001
	Pneumonia and influenza (480–488)	0.36±2.34	0.14±0.78	<0.001
	Chronic obstructive pulmonary disease and allied conditions (490–496)	0.37±1.09	0.09±0.84	<0.001
	Pleurisy (511)	0.02±0.20	0.00±0.02	0.010
	Pneumonitis (515–517)	0	0	NA
**Diseases of the digestive system (520–579)**		5.78±6.99	2.66±5.03	<0.001
	Disease of oral cavity, salivary glands and jaws (520–529)	3.26±3.49	1.60±3.35	<0.001
	Disease of esophagus, stomach, and duodenum (530–537)	1.20±3.22	0.54±2.50	<0.001
	Noninfectious enteritis and colitis (555–558)	0.52±1.42	0.22±0.85	<0.001
	Other diseases of intestines and peritoneum (560–569)	0.33±1.17	0.22±1.94	<0.001
	Liver diseases (570–573)	0.36±1.67	0.06±0.46	<0.001
	Diseases of gall bladder/biliary tract/pancreas (574–577)	0.10±0.08	0.01±0.22	<0.001
**Diseases of the musculoskeletal system and connective tissue (710–739)** *		4.35±7.48	1.22±4.21	<0.001
	Athropathies (711–719)	1.87±4.45	0.33±1.84	<0.001
	Spine disorders (720–724)	0.73±2.16	0.49±2.10	<0.001
	Disorder of tendon, brusa, muscle and fascia (725–729)	1.08±2.63	0.36±1.57	<0.001
	Osteopathies and chondropathies (730–733)	0.15±1.11	0.02±0.19	<0.001
**Diseases of the skin and subcutaneous tissue (680–709)**		3.89±5.90	0.91±2.84	<0.001
	Infection of skin and subcutaneous tissue (680–686)	0.51±1.67	0.09±0.66	<0.001
	Inflammatory conditions of skin and subcutaneous tissue (690–698)	2.24±4.21	0.41±1.70	<0.001
	Other diseases of skin and subcutaneous tissue (700–709)	1.15±2.35	0.40±1.73	<0.001
**Disorders of the eye and adnexa (360–379)**		2.01±3.38	0.68±2.46	<0.001
**Diseases of female genital organ (610–629)** †		1.93±4.45	1.11±3.39	0.001
**Diseases of the circulatory system (390–459)**		1.68±5.37	0.63±3.09	<0.001
**Diseases of the blood and blood-forming organs (280–289)**		1.22±4.61	0.04±0.53	<0.001
**Endocrine, nutritional and metabolic diseases, and immunity disorders (240–279)**		1.22±3.78	0.45±2.51	<0.001
**Infectious and parasitic diseases (001–139)**		0.99±2.56	0.37±1.71	0.001
**Diseases of kidney (580–589)**		0.82±3.82	0.08±1.47	<0.001
**Mental disorders (290–319)**		0.80±3.13	0.37±2.67	<0.001
**Diseases of urinary system (590–599)**		0.59±2.18	0.21±1.06	<0.001
**Neoplasms (140–239)**		0.55±2.95	0.26±1.79	<0.001
**Diseases of the nervous system (320–359)**		0.53±2.20	0.13±0.97	<0.001
**Diseases of the ear and mastoid process (380–389)**		0.22±0.76	0.19±1.11	<0.001
**Complications of pregnancy, child birth, and the puerperium (630–679)** †		0.17±1.02	0.11±0.80	0.497
**Diseases of male genital organ (600–608)** ‡		0.09±0.45	0.13±0.99	0.497
**Number of organ system involved** §				
	1	3 (0.9)	49 (3.6)	0.007
	2	9 (2.7)	88 (6.5)	0.010
	≥3	324 (96.1)	595 (44.1)	<0.001

*Excluding the ICD-9-CM code 710.0.

† Female only (patients with SLE, n = 293; controls, n = 1,172).

‡ Male only (patients with SLE, n = 44; controls, n = 176).

SD, standard deviation; SLE, systemic lupus erythematosus; NA, not applicable.

§ Represented as number (%).

Among the disorders of the respiratory systems, the frequencies for ambulatory visits for acute respiratory infections (ICD-9-CM codes 460–466), other diseases of the upper respiratory tract (ICD-9-CM codes 470–478), pneumonia and influenza (ICD-9-CM codes 480–488), chronic obstructive pulmonary disease and allied conditions (ICD-9-CM codes 490–496), and pleurisy (ICD-9-CM codes 511) were significantly higher in patients with SLE. Among the disorders of the digestive systems, ambulatory visits for disease of oral cavity, salivary glands, and jaws (ICD-9-CM codes 520–529), diseases of the esophagus, stomach, and duodenum (ICD-9-CM codes 520–529), noninfectious enteritis and colitis (ICD-9-CM codes 555–558), other diseases of intestines and peritoneum(ICD-9-CM codes 560–569), liver diseases (ICD-9-CM codes 570–573), and diseases of gall bladder/biliary tract/pancreas (ICD-9-CM codes 574–577) were significantly more frequent in patients with SLE. Among the diseases of the musculoskeletal system and connective tissue (710–739, but excluding SLE), the frequencies for ambulatory visits for arthropathies (ICD-9-CM codes 711–719), spine disorders (ICD-9-CM codes 720–724), disorder of tendon, brusa, muscle and fascia (ICD-9-CM codes 725–729), and osteopathies and chondropathies (ICD-9-CM codes 730–733) were significantly higher in patients with SLE. Among the diseases of the skin and subcutaneous tissue, ambulatory visits for infection of skin and subcutaneous tissue (ICD-9-CM codes 680–686), inflammatory conditions of skin and subcutaneous tissue (ICD-9-CM codes 690–698), and other diseases of skin and subcutaneous tissue (ICD-9-CM codes 700–709) were significantly more frequent in patients with SLE. In addition, most of the patients with SLE had already suffered from three or more organ systems disorders compared with controls (96.1% vs. 44.1%, *P*<0.001) during the 0.5 to 2 years preceding the index date.

### Percentages of ambulatory medical care utilization in patients with SLE and controls for four common systemic disorders over the eight-year study period

**Figure 1:**
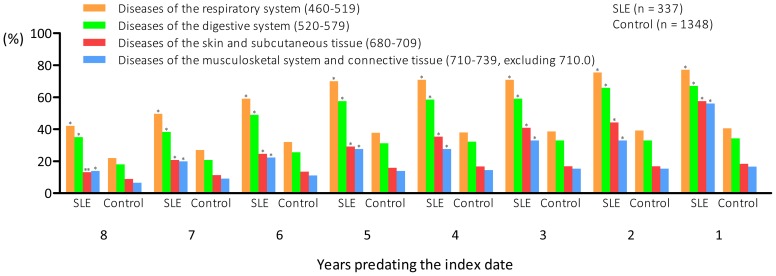
Annual percentages of ambulatory medical care visits for four common systemic disorders in patients with systemic lupus erythematosus and controls over an eight-year period preceding the index date.

We investigated the annual percentages of the four most common disorders leading to ambulatory medical care utilization in patients with SLE during the pre-SLE period. We found that the percentages of patients with SLE suffering from these four diseases were all significantly higher than controls over the study period. Moreover, the differences increased the closer the year was to the index date (Fig. 1). One year before the diagnosis of SLE, 77.2% of the patients with SLE suffered from diseases of the respiratory system, 67.1% from diseases of the digestive system, 53.4% from diseases of the musculoskeletal system and connective tissue (excluding SLE), and 57.6% from diseases of the skin and subcutaneous tissue.

### Percentages of patients who had received medical tests that might be associated with clinical manifestations of SLE

**Figure 2:**
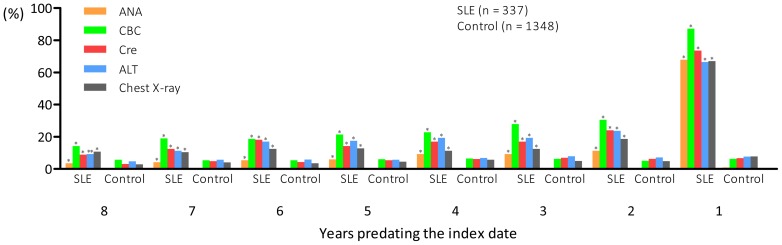
Annual percentages of antinuclear antibodies (ANA) test (via indirect immunofluorescence assay), complete blood count (CBC) test, creatinine (Cre) test, alanine aminotransferase (ALT) test, and chest X-ray from ambulatory medical care services patients with systemic lupus erythematosus and controls over an eight-year period preceding the index date. *P<0.001, **P = 0.002.

Annual percentages of medical tests performed including ANA, CBC, Cre, ALT and chest X-ray over the eight-year period preceding the index date were calculated. Although patients with SLE had received these tests more frequently compared with controls during the entire eight-year study period, most of the patients with SLE only received these tests within the one year period before the index date (Fig. 2).

### Percentages of hospitalization in patients with SLE and controls over the eight-year study period

Even after tracing back as far as eight years before the index date, there were still a small but significantly higher percentages of hospitalization in patients with SLE compared with controls (7.4% vs. 3.3%, *P*<0.001) (Table 6). The differences were similar over the entire study period except one year prior to the index date where the largest difference was observed between patient with SLE and controls (49.6% vs. 4.1%, *P*<0.001).

**Table 6:** Number of patients with systemic lupus erythematosus and controls hospitalized over an eight-year period preceding the index date.

Years preceding index date	Group, number (%)		*P*
	Patients with SLE	Controls	
	n = 337	n = 1,348	
8	25 (7.4)	45 (3.3)	**<0.001**
7	28 (8.3)	53 (3.9)	**<0.001**
6	30 (8.9)	52 (3.9)	**<0.001**
5	32 (9.5)	54 (4.0)	<0.001
4	36 (10.7)	63 (4.7)	<0.001
3	34 (10.1)	56 (4.2)	<0.001
2	30 (8.9)	70 (5.2)	<0.001
1	167 (49.6)	55 (4.1)	<0.001

SLE, systemic lupus erythematosus.

### Percentages of rheumatologist consultation before the index date

Although there were small but significantly higher percentages of consultations to rheumatologist in patients with SLE eight years predating the index date compared with controls, only 56.4% of the patients with SLE had ever consulted a rheumatologist within one year preceding the diagnosis of SLE (Table 7). In contrast, just one year after the definitive diagnosis of SLE, the percentage of consultations to a rheumatologist increased drastically to 85.8% (Table 7).

**Table 7:** Number of patients with systemic lupus erythematosus and controls who had visited a rheumatologist over an eight-year period preceding the index date.

Years preceding index date	Group, number (%)		*P*
	Patients with SLE	Controls	
	n = 337	n = 1,348	
8	25 (7.4)	33 (2.4)	<0.001
7	22 (6.5)	38 (2.8)	<0.001
6	34 (10.1)	45 (3.3)	<0.001
5	39 (11.6)	51 (3.8)	<0.001
4	42(12.5)	62 (4.6)	<0.001
3	47 (13.9)	59 (4.4)	<0.001
2	46 (13.6)	57 (4.2)	<0.001
1	190 (56.4)	61 (4.5)	<0.001
One year after index date	289 (85.8)	114 (8.4)	<0.001

SLE, systemic lupus erythematosus.

## Discussion

Using a nationwide, population-based health claim database, we reported a systematic analysis of clinical course of SLE disorders predating the diagnosis of SLE. To our knowledge, this is the first overall analysis of the clinical course of SLE conducted in an Asia population. Previous studies have reported the very early clinical course of SLE but most of them had focused on the appearance of autoantibody [16]. A study on 130 U.S. patients showed that the earliest symptoms associated with the ACR criteria for SLE appeared before the diagnosis were discoid rash and seizures, with a mean onset of 1.7 years before diagnosis. Other symptoms associated with the ACR criteria for SLE including arthritis, skin disorders, hematological disorders, serositis, and renal diseases were also commonly presented prior to the diagnosis of SLE [4]. Moreover, thrombotic events were reported to increase in patients with SLE prior to diagnosis [10]. In addition, a Swedish case-control study of 38 patients with SLE found that ACR defined SLE criteria including arthritis, skin disorder, and serositis appeared prior to diagnosis of SLE [7].

Our study showed that patients with SLE already suffered from diseases of the respiratory, digestive, and musculoskeletal systems and skin tissue (Fig. 2) and multiple system disorders (Table 5) several years preceding the definitive diagnosis of SLE. In addition, most of the patients with SLE only received relevant medical tests just within a one-year period prior to the diagnosis of SLE. ANA is a useful test for the initial differential diagnosis of SLE. We found that only 68% of the patients with SLE had ever received an ANA test from ambulatory medical care services while the remaining patients received the test only during hospitalization. A previous study conducted in Taiwan indicated that the positivity rate of ANA in patients with SLE was over 90% [17]. According to the study by Wichainun et al. [18], the ANA test has a sensitivity of over 90% to detect SLE, which is considerably higher than those from other autoantibodies such as anti-dsDNA and anti-SSA/SSB [19]. Therefore, ANA test is a test of choice for initial screening of SLE among patients with multisystemic disorders. Clinicians should consider the possibility of SLE and arrange screening tests such as ANA test when young female patients are presented with multiple systemic disorders.

We noted that patients with SLE visited ambulatory medical services more frequently as a result of a wide array of different clinical disorders. Diseases of the respiratory systems were the most frequent cause of visit for ambulatory medical care. Chronic interstitial lung diseases, acute lupus pneumonitis, or diffuse alveolar hemorrhage [20], and viral, bacterial, or fungal respiratory tract infection related to impaired immunity [21] are commonly noted in patients with SLE. We found various disorders of the respiratory system increased the ambulatory medical utilization in patient with SLE during pre-SLE period. It should be noted that although pleuritis is one of the ACR criteria for SLE, it was rarely reported as a diagnosis during the 0.5 to 2 years preceding the diagnosis of SLE in our study.

For the gastrointestinal disorders, oral ulcer is a common initial presentation of SLE. Other common gastrointestinal disorders associated with SLE itself or the consequence of the medication such as dysphagia, anorexia, nausea, vomiting, hemorrhage, hepatitis, and abdominal pain [22], [23] might explain the increased frequency of visit for diseases of the digestive system. Skin and musculoskeletal disorders are both well-known clinical symptoms preceding the diagnosis of SLE [4], [7] and common initial presentation of SLE [24], [25]. Our findings were consistent with previous reports.

Regarding the diseases of the circulatory system, pericarditis is one of the ACR criteria of SLE but myocarditis, pulmonary hypertension, and valvular heart disease also belong to cardiac involvement of SLE. The ICD-9-CM codes for circulatory diseases (390–459) contain venous thrombosis and vasculitis. The former is well known to develop in patients with SLE prior to the diagnosis [10] and the latter is frequently observed in patients with SLE. Previous studies [4], [10] showed that hematological system (such as thrombocytopenia, leucopenia, and lymphopenia) and central nervous system disorders were often noted in patients with SLE predating the diagnosis. Our study revealed similar findings. Furthermore, the results of increased ambulatory medical visits for renal disorder in patients with SLE predating the diagnosis were in line with findings from previous studies [4], [7].

Somewhat to our surprise, we found patients with SLE visits ambulatory medical care services more frequently than controls in clinical disorders that are not among the diseases associated with the ACR criteria for classification of SLE. Ocular manifestations in SLE are fairly common, and almost any part of the eye and visual pathway can be affected by SLE. Common ocular manifestations of SLE, including dry eye, scleritis, episcleritis, optic neuritis, retinopathy, and uveitis, may appear as the initial presentation of SLE [24], [26]. For the endocrine system, the prevalence of autoimmune thyroid diseases is higher in patients with SLE [27], [28] and this could explain our findings. The frequencies of visits for disease of female genital organ, urinary tract, and the ear were also found to be elevated in patients during the pre-SLE period. Although these organ systems could directly be affected by SLE [29], [30], the increased visits might also be caused by infections of these organs. A study based on the Taiwan NHI database demonstrated an increased risk of tuberculosis in patients before their diagnosis of SLE [31]. Nevertheless, the risks of other infectious diseases remain to be investigated. Frequencies of visits for neoplasm were also elevated in patients during the pre-SLE period. Patients with SLE had elevated risk for developing cancer late in the diseases course [32] and a concordance development of the SLE and cancer has also been reported [33]. Moreover, the ICD-9-CM codes for neoplasm (140–239) contain not only malignant tumors but also benign tumors. Therefore, additional studies will be needed to clarify this finding.

Findings from this study also showed that many disorders occurred earlier and in a more complex fashion compared with the results from previous studies [4], [6]. Such finding might be attributed to the unique medical system in Taiwan and therefore, our results might not be generalizable to other countries. Under the Taiwan's NHI scheme, the out-of-pocket expenses paid by patients for a single ambulatory visit ranges from just US$1.7 in local clinics to US$18.6 in medical centers. The number of annual physician consultations per capita was 13.4 in Taiwan, which was higher than any country in the Organisation for Economic Co-operation and Development (OECD) [34]. In addition, patients in Taiwan can directly consult specialists without the need for a referral from a general practitioner. Therefore, it was not surprised to find that 44.1% of the controls had ambulatory medical visits due to three or more organ system disorders. Chen et al. [14] demonstrated that 79.8% of the general population had consulted more than two specialists within one year. Our study also showed that despite the high frequencies of ambulatory medical visits and the presence of a variety of clinical disorders in patients during their pre-SLE period, relatively few of them had ever consulted a rheumatologist. Even up to just one year preceding the diagnosis of SLE, only about a half of the patients had a rheumatologist consultation. More effort should be put into public health education to increase the public's awareness of SLE and health care providers should consider the possibility of SLE when young female patients are presented with disorders involving multiple organs. Moreover, it is critical to identify pre-SLE patients as early as possible, for example, through the use of questionnaire [35] and screening tools such as the ANA test. Timely treatment for those at elevated risks of developing SLE might help to reduce the high mortality associated with SLE [36].

Several limitations of this study merit attention. First, we were unable to confirm that the application date of the catastrophic illness certificate for SLE was the same date that a patient received his or her definitive diagnosis of SLE. To estimate the lag time between the application date of the catastrophic illness certificate and the definitive diagnosis of SLE, we took the date of the last three consecutive visits with diagnosis of SLE in a given patient as the date of his or her definite diagnosis of SLE. Based on this calculation, we found a mean lag time of only 11.9 days (SD = 29.1 days). Therefore, this relatively short lag time should not materially affect our results. In addition, the diagnosis of SLE in our study was based on the 1997 ACR revised criteria for the classification of SLE [13]. The adoption of the 2012 Systemic Lupus International Collaborating Clinics classification criteria [37] may be able to shorten the period between the onsets of clinical disorders to the final diagnosis of SLE [38]. Furthermore, due to the limitations of our data source, no radiographic reports, serological data (including the complements and autoantibodies), and family history are available.

In conclusion, in this national, population-based study, we found significant increased frequencies and costs of annual ambulatory medical care utilization in SLE patients over a period of at least eight years prior to their definitive diagnosis of SLE. Over the 0.5 to 2 years period preceding the diagnosis of SLE, the frequencies of medical visits related to almost all organ systems were significantly higher in patients with SLE compared with controls. However, only a half of the pre-SLE patients had ever consulted a rheumatologist within one year before their definitive diagnosis of SLE. More effort should be put towards facilitating the recognition of SLE in its early stage.
